# Shelf Life Determination of Fresh Blueberries (*Vaccinium corymbosum*) Stored under Controlled Atmosphere and Ozone

**DOI:** 10.1155/2015/164143

**Published:** 2015-01-15

**Authors:** Anibal Concha-Meyer, Joseph D. Eifert, Robert C. Williams, Joseph E. Marcy, Gregory E. Welbaum

**Affiliations:** ^1^Food Science and Technology Department, Virginia Tech, 1230 Washington Street SW, Blacksburg, VA 24061, USA; ^2^Centro de Estudios en Alimentos Procesados (CEAP), Avenida San Miguel 3425, 3480137 Talca, Chile; ^3^Horticulture Department, Virginia Tech, 1880 Pratt Drive, Research Building XV, Blacksburg, VA 24061, USA

## Abstract

Fresh blueberries are commonly stored and transported by refrigeration in controlled atmospheres to protect shelf life for long periods of storage. Ozone is an antimicrobial gas that can extend shelf life and protect fruit from microbial contamination. Shelf life of fresh highbush blueberries was determined over 10-day storage in isolated cabinets at 4°C or 12°C under different atmosphere conditions, including air (control); 5% O_2_ : 15% CO_2_ : 80% N_2_ (controlled atmosphere storage (CAS)); and ozone gas (O_3_) 4 ppm at 4°C or 2.5 ppm at 12°C, at high relative humidity (90–95%). Samples were evaluated for yeast and molds growth, weight loss, and firmness. CAS and O_3 _did not delay or inhibit yeast and molds growth in blueberries after 10 days at both temperatures. Fruit stored at 4°C showed lower weight loss values compared with 12°C. Blueberries stored under O_3_ atmosphere showed reduced weight loss at 12°C by day 10 and loss of firmness when compared to the other treatments. Low concentrations of ozone gas together with proper refrigeration temperature can help protect fresh blueberries quality during storage.

## 1. Introduction

Blueberries are recognized for their contribution to a healthy diet with different beneficial bioactive compounds such as flavonoids, anthocyanins, and others [1, 2], which helps to avoid important diseases including different cancers [3, 4]. Highbush blueberries (*Vaccinium corymbosum*) together with the other commercial blueberry species are ranked as the second most economically important berry after strawberries in the U.S., accounting for nearly 850.9 million dollars in 2012 [5]. Although the U.S. is the world biggest blueberry producer, the American market is so large that importing the fruit from other countries such as Chile is required. In 2011, Chile provided more than 50% of the imported blueberries sold in the U.S. [6]. Products shipped from Chile can take 20 days to arrive to the U.S. [7].

Proper storage for blueberries is around 0°C, with a relative humidity from 90 to 95% that provides a storage life of 10–18 days [8]. The use of controlled atmosphere in fresh produce transportation is widely applied by producers, to assure the quality of the product and avoid spoilage. Respiration rate as well as deterioration decreases for some fruits when under CO_2_ levels of about 10% to 20%. Bounous et al. [9] reported that O_2_ concentrations between 8% and 10% and CO_2_ concentrations of 10% to 13% managed to maintain the quality of blueberries between 5 and 8 weeks at 0-1°C and 3 days at 18–20°C. According to Retamales et al. [10] high levels of CO_2_ (20–30 kPa) in combination with low levels of O_2_ (5–10 kPa) helped control decay and extend shelf life of cherries transported from Chile to Japan. Moreover, reduction of* Botrytis cinerea* (grey mold rot) in strawberries can be achieved by applying 15% CO_2_ [11]. According to Mitcham and Mitchell [12] grey mold rot and other decay organisms can be minimized by using gas concentrations of 15 to 20% carbon dioxide and 5 to 10% oxygen, which will also decrease respiration and softening rates of shipped blueberries, raspberries, and blackberries thus prolonging postharvest life.

Ozone (O_3_) is a strong antimicrobial agent with variety of applications in the food industry [13]. Ozone was designated as generally recognized as safe (GRAS) in 1982 by the U.S. Food and Drug Administration (FDA) for use as a disinfectant or sanitizer in the gas or liquid phase on food (21 CFR, Part 173) and for direct contact use as an antimicrobial for treatment, storage, and processing on diverse foods including raw and minimally processed fruits and vegetables [14]. Ozone decomposes rapidly into oxygen without leaving residues [15] and its postharvest applications have increased [16]. Moreover, its use in cold rooms helps reduce the ethylene (C_2_H_4_) level in air, extending the storage life of fruits and vegetables such as apples and oranges [17]. Other studies have proven inhibition of spoilage microorganisms and shelf life extension using O_3_ in bananas [18], potatoes, onions and beetroot [19], tomatoes and mandarins [20], blackberries [21], lettuce and carrots [22], and strawberries [23].

However, other studies have found ozone to be ineffective to control spoilage on apples, blueberries, green beans, muskmelons, peaches, and strawberries [24]. Furthermore, apples, cantaloupes, cranberries, and corn kernels showed increased decay and spoilage contamination when ozone was used on them [25–27].

According to Palou et al. [28] 0.3 ppm O_3_ on peaches and table grapes did not delay decay; however grey mold was inhibited on grapes. Pérez et al. [23] found 0.35 ppm O_3_ inefficient to prevent fungal decay in addition to negative effects on the sensory properties of strawberries. Although high O_3_ may be necessary for an effective elimination of microorganisms, this may alter negatively the sensory attributes of fresh fruit [29].

The present study evaluated shelf life extension and quality preservation of fresh blueberries packaged under different atmosphere conditions (Air; controlled atmosphere storage (CAS): 5% O_2_ : 15% CO_2_ : 80% N_2_; or Ozone) and temperature storage (4°C or 12°C) for 10 days.

## 2. Materials and Methods

### 2.1. Fruit Samples

The trial was carried out during the summer of 2012 using highbush blueberries (*Vaccinium corymbosum* L., cv Ozark Blue) that were hand harvested from the commercial planting Three Birds Berry Farm located in Blacksburg, VA. Two replications were conducted each consisting of fruit harvested at two different dates. Only fully colored developed fruit without defects was selected on a visual basis.

### 2.2. Fruit Storage

After harvest collection, samples were stored in a cooler (4–8°C) and transported to the Food Science and Technology Building at Virginia Tech. Samples consisted of fifty blueberries stored in 4.4 oz PET perforated retail clamshell boxes (Highland Corporation, Mulberry, FL), which were placed in a dessicator cabinet and incubated at different temperatures (4°C or 12°C), and different atmosphere conditions (Air as a control (21% O_2_ and 0.03% CO_2_); 5% O_2_ : 15% CO_2_ : 80% N_2_; Ozone). Ten samples were analyzed on days 0, 1, 4, 7, and 10 per storage temperature and atmosphere conditions.

### 2.3. Controlled Atmosphere and Ozone Treatment

Clear acrylic desiccator cabinets with exterior dimensions of 12′′W × 12′′H × 12′′D with gas ports including hygrometer (Cole-Parmer, Lansing, MI, USA) were used to store fruit in clamshells, inside an incubator (Precision Incubator, Thermo Scientific, Waltham, MA) and held at 4°C or 12°C with 90–95% relative humidity (RH), which was maintained placing a deionized water in an open sterilized petri dish inside the desiccators. Temperature and RH were monitored by a sensor (Traceable Jumbo Thermo-Humidity Meter, Fisher Scientific, Pittsburgh, PA).

For controlled atmosphere storage a certified standard-spec compressed gas tank (MID-Saint Louis SGL-MO) supplied an atmosphere of 5% O_2_ and 15% CO_2_ and balanced with nitrogen (80%). Gas concentrations were maintained by reflushing the cabinets after monitoring them two times a day (morning and late afternoon) using an O_2_/CO_2_ gas analyzer (O_2_/CO_2_ Check Point, PBI-Dansensor, Ringsted, Denmark).

A corona discharge ozone generator (FreshFridge 2.0 Refrigerator Air Purifier Model GH 2138, IonCare GandH Industrial Ltd., China) and a small air circulating fan were installed in the chambers. Ozone concentration in chambers was monitored, controlled, and recorded continuously using an ozone analyzer (Model ES-600, Ozone Solutions Inc., IA, USA) and a Dell Latitude D830 computer as data logger (Dell, Round Rock, Texas). Ozone concentration mean and standard deviation values were 4.0 ± 1.8 ppm for 4°C and 2.5 ± 1.5 ppm at 12°C. A third chamber without an ozone generator served as the control (Air). Blueberries were exposed to controlled atmosphere and ozone in these chambers for a total of 10 days.

### 2.4. Weight Loss

Each clamshell with fifty blueberries was weighed after harvest and the reweighed after 1, 4, 7, and 10 days of storage. Weight was recorded using a scale with an accuracy of 0.01 g and expressed as accumulated weight loss percentage per unit time.

### 2.5. Yeast and Molds

Yeast and Molds Petrifilm (3M Microbiology Products, St. Paul, MN) pouches were stored unopened at 4°C. To aseptically prepare 1 : 10 dilutions, 10 g of blueberries was added to a stomacher bag with 90 mL of sterile 0.1% peptone water (Difco, Becton Dickinson, Sparks, MD). Samples were blended and stomached for 120 seconds (AES Laboratoire Easy Mix, Microbiology International, Combourg, France), serially diluted and plated on Yeast and Mold Petrifilm in duplicate. Plates were incubated in horizontal position, clear side up, in stacks not exceeding 20 units for 3 days at room temperature 20–25°C.

Yeasts appeared as blue-green or off-white in color and formed small defined edge colonies. Mold colonies are usually blue but may also assume their natural pigmentation (e.g., black, yellow, and green). They tended to be larger and with more diffuse edges than yeast colonies, usually with a focus in a center of colony. Yeast and molds were enumerated using the AOAC official method 997.02 [30].

### 2.6. Firmness Texture Analysis

Textural measurements were performed on harvest day and during postharvest storage. Blueberries samples for analysis were randomly selected and sample size was fifteen berries each testing, as described by Døving et al. [31]. The force required to penetrate each blueberry fruit was measured individually using a TA.XT*Plus* Texture Analyzer and a 2 mm diameter stainless steel puncture probe TA-52 (Stable Microsystems, Godalming, Surrey, UK). The probe height was calibrated to 13 mm above the TA-90 base platform so that the blueberry could be aligned directly under the probe and a 50 Kg load transducer was used. The following test settings were used: Measure Force in Compression; Return to Start; pretest speed of 2 mm/s and test speeds of 1 mm/s with an automatic trigger set to 5 grams of force; and test distance of 3 mm into the blueberries. Each time a set of fruits was measured, the equipment was force and height calibrated.

### 2.7. Experimental Design and Statistical Analysis

Data were analyzed using the least squares method on JMP Pro 10 (SAS Institute Inc., Cary, NC). The randomized complete block factorial design with two replications was utilized to test the treatments and their interactions on weight loss, yeast and mold growth, and texture firmness. If the interactions between treatments were not significant (*P* > 0.05), the main effects of the treatments were separated using Student's *t*-test.

## 3. Results and Discussion

### 3.1. Weight Loss

**Figure 1:**
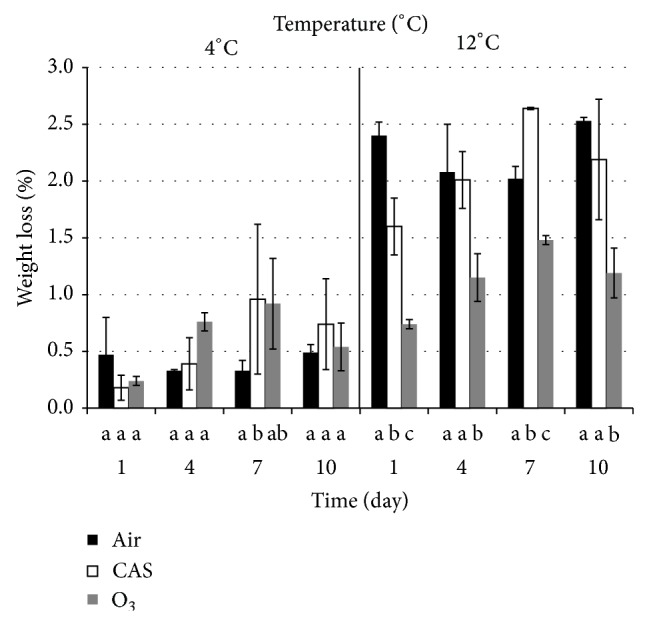
Weight loss percentage of highbush blueberries stored at 4°C or 12°C under controlled atmosphere storage conditions (CAS), ozone (O_3_), or regular atmosphere (Air) during 10 days. Columns within each day at same temperature followed by the same lowercase letter are not significantly different (*P* > 0.05) from each other.

Although weight loss was minimal in this study and ranged from 0.18 to 2.64% within 10 days of storage, significant differences in treatments were observed mostly at 12°C storage. Weight loss of fruit stored at 4°C showed no significant difference among treatments or time of storage (Figure 1). According to literature, to control weight loss of blueberries it is crucial to keep low temperatures (0-1°C) and high relative humidity during storage [32, 33]. Forney [34] found that maintaining a high relative humidity (95% or greater) minimized weight loss and shrivel of blueberries. However, this can increase decay development if condensation is not properly controlled [35]. Schotsmans et al. [36] concluded that controlled atmosphere storage (2.5 kPa O_2_ + 15 kPa CO_2_) was favorable for long term blueberry storage up to 42 days. Furthermore, a significant weight loss (9–14%) was not observed until day 6 of exposure and the antioxidant activities and total phenolic content of blueberries were not affected adversely [36].

Despite the fact that in the present study the same ozonator device was used at 4°C and 12°C, ozone gas concentration averaged 4 ppm and 2.5 ppm, respectively, in agreement with Liew and Prange [24] who observed that residual ozone concentrations are lower at higher storage temperatures. At a storage temperature of 12°C, O_3_ was the treatment that better protected the fruit from weight loss over time and values were significantly lower than weight loss under air and CAS at days 1, 4, 7, and 10 (Figure 1). Sanford et al. [33] reported that maximum weight loss before blueberries become nonsaleable is approximately 5% to 8%, implying that in this study there was not a loss of quality since values were far from this range. The results in this study are equivalent to other research that showed around 2% loss from initial weight after 10 days of storage at 5°C and 10°C [37] and minimal weight loss of 2% after 14 days at 0°C [38].

O_3_ kept weight loss low and helped control decay at 12°C, while other authors found reciprocal results on stored highbush blueberries at 10°C for 7 days using 700 ppb and no phytotoxicity compounds were observed [39]. According to Kim et al. [40] SO_2_ and O_3_ reduce weight loss and fruit decay in blueberries increasing fruit quality and storage life.

In the present study by day 10, CAS and air showed no significant difference in weight loss at 12°C, implying that CAS is effective controlling weight loss in a long time storage period. Although Bounous et al. [9] observed that three cultivars of highbush blueberries lost more weight when stored in air and O_3_ when compared to CAS, at the end of 6 weeks storage, CAS weight loss was higher than air and O_3_.

Weight loss in fruit is directly related to respiration rate [38]. A low O_2_ and rich CO_2_ atmosphere can potentially reduce not only the respiration rate but also ethylene sensitivity and production, oxidation, and fruit decay [41, 42]. Moreover, O_3_ inhibition of enzymatic reaction can cause a decrease in fruit respiration leading to less weight loss [40]. Other gases can produce a decrease of enzymatic activity in fruit, such as SO_2_ [40], Na_2_S_2_O_5_ sodium metabisulfite [43], and CO_2_ [9]. According to Aguayo et al. [44], O_3_ stimulated the respiration rate in both whole and cut tomatoes only during the first two days of storage, decreasing after that period. Other authors have confirmed that O_3_ decreases respiration rate in whole tomatoes [20, 45], bananas [18], and peaches [28]; however Liew and Prange [24] observed an increase on carrots under O_3_ treatments, depending on doses and storage time. The ratio of CO_2_ produced to O_2_ consumed, known as the respiratory quotient (RQ), is normally assumed to be equal to 1.0 if the metabolic substrates are carbohydrates [41]. Beaudry et al. [46] explained an observed RQ of 1.3 for blueberries by their high content of citric acid and sugars. The RQ is much greater than 1.0 when anaerobic respiration takes place. Oxygen concentration should be low but not zero, to avoid anaerobic respiration on fruit stored in controlled atmosphere conditions. In this study, oxygen concentrations under CAS were 5%.

### 3.2. Yeast and Molds

Yeast counts increased from day 0 to day 10 along all treatments and temperatures (Table 1). Initial counts in CAS and O_3_ were higher than in air. At 12°C yeast population increases were higher in O_3_ and CAS with 2.59 and 2.37 log CFU/g increases, respectively, when compared to 1.87 and 1.86 log CFU/g at 4°C. Since fruits were collected from the field and were not surface-sterilized before testing, naturally occurring yeast and molds were measured. High variability of initial yeast and mold counts is influenced by time of the year, weather, and harvest conditions, as well as the fruit wetness when picked [47]. Moreover, if the natural protective wax bloom of blueberries is absent due to weather or picking practices, it is more likely to grow yeast and molds.

Increases over time in mold counts were similar for blueberries stored in air (control) at 4°C (2.74 log CFU/g) and 12°C (2.75 log CFU/g) (Table 2). The increase over time of mold for CAS blueberries at 4°C (2.10 CFU/g) was higher compared to 12°C storage (0.20 CFU/g). Beuchat and Brackett [48] observed slower growth of molds at 10°C, when compared to 5°C, on lettuce. Lower counts at warmer temperatures were observed by day 10 on O_3_ and CAS treatments. The increase in mold counts for the O_3_ treatment at 4°C was calculated using the recovery counts of day 7, since at day 10 at 4°C no plate growth occurred at a detection limit of 10^−4^. Growth of visible mold on samples was only observed in air treatment at 12°C in day 10 (1/50 berries).

Ozone inhibition of bacteria is more noticeable than in yeast and molds [13, 44]. Moreover, Palou et al. [49] indicated that O_3_ delays but does not reduce fungi incidence after one week and cannot control molds in wounded fruits. Ozone was fungistatic against grey mold (*Botrytis cinerea*) and not fungicidal [24]. Ozone treatment was ineffective in preventing fungal decay in strawberries after 4 days at 20°C [23]. According to Schotsmans et al. [36], CA effectiveness to decrease fungal and blemish development is achieved after 28 days of storage at refrigeration conditions.

### 3.3. Firmness

Table 3 shows that firmness penetration force values were not significantly different (*P* < 0.05) between treatments after 10 days of storage at 4°C. However at 12°C, penetration force for the control berries (219.77 g) was significantly higher compared to berries stored under O_3_ (169.19 g) and CAS (182.67 g). According to Schotsmans et al. [36] a high touch firmness value means the blueberry fruit is perceived as being softer. The enhanced resistance of the fruit to the probe penetration can be interpreted as excessive elasticity or gumminess, due to a strong loss of internal water turgor pressure [50]. This characteristic is unfavorable since it can mislead fruit grading and texture determination [51]. Turgidity is the most critical texture component in blueberry [50].

At 4°C texture within O_3_ treatments did not have significant differences over time, while air only showed differences at day 1. This latter observation could be caused by heat shock from harvest temperature to the refrigeration temperatures. Aguayo et al. [44] observed that the firmness of tomato slices did not change after cyclical O_3_ enriched airflow exposure throughout 5°C cold storage time when compared to control. However, in whole tomatoes, the O_3_ treatment reduced softness. Mushrooms treated with O_3_ (0.03 mg s^−1^) for 15 or 30 min showed no significant difference in firmness change during storage [52].

At 12°C CAS blueberries showed a significant increase in texture after day 0 (196.0 g) when compared to day 1 (150.1 g) and day 4 (138.5 g). Many reports can be found in literature where strawberry firmness increased during low temperature storage [53, 54] and high CO_2_ levels [55, 56]. The firming effect of CO_2_ and its magnitude are possibly cultivar dependent [54]. Moreover, the indirect effects of CO_2_ on the apoplastic pH with the subsequent precipitation of soluble pectins and the enhancement of cell-to-cell bonding [57] are likely responsible for the firming response [58]. Higher firmness of Bluecrop blueberries during storage could also be related to the presence of stone cells in the fruit [59].

The firmness increase in CAS at 12°C when comparing day 0 (196.0 g) to day 7 (146.3 g) is equivalent to the results of the study by Mahajan and Goswami [60] where litchi fruit firmness increased, achieving acceptable puncture strength within the storage period, perhaps because of the moisture loss from litchi fruit during storage, which may be explained by fruit drying and hardening characteristics. In the present study, blueberries stored under CAS at 12°C experience high weight loss values when compared to ozone. Pelayo et al. [58] found a beneficial effect of CO_2_ during storage increased firmness in two cultivars with no detectable effects on external color.

Firmness testing of fruits is used to describe mechanical properties of the fruit tissue [61] and provides information on the storability and resistance to injury of the product during handling [62]. Instrumental measurements of texture are preferred rather than sensory evaluation since instruments may reduce variation among measurements due to human factors and are in general more precise [63]. The present study results show that the variations in texture between individual fruit are large, in agreement with previous reports [31, 62].

Texture is affected by cellular organelles, biochemical constituents, water content or turgor, and cell wall composition [64]. High humidity allows degradation of the middle lamella and disintegration of the primary cell wall, which are important factors determining fruit softening [65]. During blueberry ripening, the total water soluble pectin decreases and the degradation of the cell wall and middle lamella is responsible for the loss of firmness [65]. Changes in the chemistry of the primary cell wall components cellulose, pectins and hemicelluloses that occur during growth and development can also affect texture [66]. This variation may be attributed to cultivar differences and/or their interaction with postharvest storage conditions [67]. In the present study, the only cultivar used was Ozark Blue under high humidity environments (90–95%).

Absence of significant changes in firmness among treatments at 4°C in the present study corresponds to the results obtained by Chiabrando et al. [66] who found that, during storage, firmness of blueberries was not considered a critical quality factor. Firmness remained constant during postharvest storage at 0°C, indicating that low-temperature conditions may delay berry softening by inhibiting enzymatic activities and ethylene production.

The high variability of mean blueberry penetration force measurements can be due to the different berry sizes within the samples. Smagula et al. [68] and Khazaei and Mann [69] reported that smaller blueberries tended to be slightly firmer than larger ones (confirming the negative relation between size and firmness of blueberries from the same cultivar [36, 70]). Moreover, Døving and Måge [62] and Chiabrando et al. [66] also observed a significant amount of fruit-to-fruit variability in firmness values. Texture is not easy to define particularly in small fruits such as blueberries, since a common standardized method does not exist. Many instruments and techniques have been studied widely [62, 71–77] and the majority of these methods record a measurement of the force needed to puncture, penetrate, or deform the fruit [66]. In this study, penetration firmness measurement was conducted similar to other research with blueberries [78, 79].

At 12°C by day 10, the penetration peak force for blueberries stored in air was significantly higher on day 10 (219.8 g), compared to days 0 (178.0 g), 1 (167.6 g), and 7 (179.5 g). This increase in firmness may be attributed to moisture loss during storage, which corresponds to fruit drying and hardening characteristics [60, 66]. This behavior is due to the fact that a less turgid berry generally presents an extended tissue deformation before the probe breaks the superficial tension and enters into the plasticity phase, where irreversible rupture occurs [50]. A soft and more deformable and elastic berry structure is probably by a water leakage as reported for other products [80].

At day 10, the mean force measurement for each treatment was significantly different when compared to each other. In whole tomatoes, O_3_ treatment reduced softness after 15 days of storage, providing a better retention of the texture of fruit compared to control [44]. Daş et al. [81] observed that an ozone gas concentration of 30 mg/L did not change or soften texture of cherry tomatoes. Fan et al. [82] developed an in-package ozonation device, which produced ozone inside sealed film bags, and showed no negative effects on cherry tomatoes texture, preserving fruit quality after 22 days posttreatment storage.

At 12°C fruit firmness increased initially in all treatments when comparing day 0 and day 1, while firmness decreased over storage time, in agreement with results reported by Allan-Wojtas et al. [59], Chiabrando et al. [66], and Basiouny and Chen [83] in blueberries, and Pelayo et al. [58] in strawberries. Shriveling of blueberries over time was observed regardless of storage temperature in agreement with other authors [37, 66].

## 4. Conclusion

Ozone was more effective controlling weight loss at 12°C, although weight loss in fruit was never more than 3%. A high weight loss in fruit is a problem that is commercially significant for producers. Naturally occurring yeast and molds on blueberries were not affected by CAS and O_3_ treatments, since they were able to grow over time at both temperatures. During storage time, firmness was better maintained with O_3_ and CAS treatment when compared to Air. These treatments can be used during cold storage, meaning an advantage to producers who may wish to delay or extend marketing of fruits [84]. Ozone treatment did not cause external damage to the blueberries. The O_3_ treatment reported here can be used in fresh highbush blueberries to maintain quality and extend shelf life. Future research could study the impact of O_3_ and CAS on flavor profiles using a sensory panel.

## Figures and Tables

**Table 1:** Yeast counts (log CFU/g) and increase over 10 days (Δ) of highbush blueberries stored at 4°C or 12°C under controlled atmosphere storage conditions (CAS), ozone (O_3_), or regular atmosphere (Air).

Treatment	4°C			12°C		
	Day 0	Day 10	Δ	Day 0	Day 10	Δ
Air	3.01 ± 0.10	3.95 ± 0.27	0.94	3.01 ± 0.10	3.93 ± 0.15	0.92
CAS	5.43 ± 0.05	7.30 ± 0.10	1.87	3.83 ± 0.30	6.42 ± 0.03	2.59
O_3_	5.44 ± 0.05	7.29 ± 0.14	1.86	3.77 ± 0.36	6.14 ± 0.06	2.37

**Table 2:** Mold counts (log CFU/g) and increase over 10 days (Δ) of highbush blueberries stored at 4°C or 12°C under controlled atmosphere storage conditions (CAS), ozone (O_3_), or regular atmosphere (Air).

Treatment	4°C			12°C		
	Day 0	Day 10	Δ	Day 0	Day 10	Δ
Air	<1.00^*^	3.74	2.74	<1.00^*^	3.75	2.75
CAS	2.85	4.95	2.10	2.24	2.60	0.20
O_3_	2.86	4.74^**^	1.89	2.24	3.39	1.15

^*^No growth at limit of detection.

^**^Mold counts at day 7 used, because of no growth at day 10 due to high limit of detection.

**Table 3:** Firmness penetration peak force (g) of highbush blueberries stored at 4°C or 12°C under controlled atmosphere storage conditions (CAS), ozone (O_3_), or regular atmosphere (Air) during 10 days.

Firmness penetration peak force (g)					
Treatment	Day 0	Day 1	Day 4	Day 7	Day 10
Temperature 4°C					
Air	178.0 ± 42.5^Aa^	138.8 ± 23.7^Ba^	180.8 ± 17.5^Aa^	195.3 ± 46.3^Aa^	185.6 ± 34.3^Aa^
CAS	159.2 ± 24.7^CDa^	183.0 ± 26.0^ABb^	140.5 ± 20.9^Db^	167.8 ± 36.2^BCa^	194.4 ± 44.2^Aa^
O_3_	159.2 ± 24.7^Aa^	169.0 ± 26.6^Ab^	170.1 ± 33.8^Aa^	176.1 ± 39.1^Aa^	173.4 ± 51.7^Aa^

Temperature 12°C					
Air	178.0 ± 42.5^Aa^	167.6 ± 23.3^Aa^	192.0 ± 17.5^Aa^	179.5 ± 23.4^Aa^	219.8 ± 57.3^Ba^
CAS	196.0 ± 28.9^Aab^	150.1 ± 26.8^Bb^	138.5 ± 21.9^Bb^	146.3 ± 18.4^Bb^	182.7 ± 35.5^Ab^
O_3_	202.8 ± 24.8^Ab^	146.1 ± 15.1^Bb^	153.1 ± 15.6^Bc^	143.5 ± 16.1^Bb^	169.2 ± 26.6^Cb^

Means within each row followed by the same capital letter are not significantly different (*P* > 0.05) from each other. Means within each column followed by the same lowercase letter are not significantly different (*P* > 0.05) from each other.
